# The role of RND-type efflux pumps in multidrug-resistant mutants of *Klebsiella pneumoniae*

**DOI:** 10.1038/s41598-020-67820-x

**Published:** 2020-07-02

**Authors:** Rui Ting Ni, Motoyasu Onishi, Minako Mizusawa, Ryoko Kitagawa, Takanori Kishino, Futoshi Matsubara, Tomofusa Tsuchiya, Teruo Kuroda, Wakano Ogawa

**Affiliations:** 1 Department of Microbiology, Graduate School of Medicine, Dentistry and Pharmaceutical Sciences, Okayama University, Okayama, 700-8530 Japan; 2 Department of Microbiology, Faculty of Pharmaceutical Sciences, Okayama University, Okayama, 700-8530 Japan; 3 Department of Microbiology and Biochemistry, Daiichi University of Pharmacy, Fukuoka, 815-8511 Japan; 4 Department of Microbiology, Graduate School of Biomedical and Health Sciences, Hiroshima University, Hiroshima, 734-8553 Japan

**Keywords:** Bacteria, Bacteriology, Antimicrobial resistance

## Abstract

The emergence of multidrug-resistant *Klebsiella pneumoniae* is a worldwide problem. *K. pneumoniae* possesses numerous resistant genes in its genome. We isolated mutants resistant to various antimicrobials in vitro and investigated the importance of intrinsic genes in acquired resistance. The isolation frequency of the mutants was 10^−7^–10^−9^. Of the multidrug-resistant mutants, hyper-multidrug-resistant mutants (EB256-1, EB256-2, Nov1-8, Nov2-2, and OX128) were identified, and accelerated efflux activity of ethidium from the inside to the outside of the cells was observed in these mutants. Therefore, we hypothesized that the multidrug efflux pump, especially RND-type efflux pump, would be related to changes of the phenotype. We cloned all RND-type multidrug efflux pumps from the *K. pneumoniae* genome and characterized them. KexEF and KexC were powerful multidrug efflux pumps, in addition to AcrAB, KexD, OqxAB, and EefABC, which were reported previously. It was revealed that the expression of *eefA* was increased in EB256-1 and EB256-2: the expression of *oqxA* was increased in OX128; the expression of *kexF* was increased in Nov2-2. It was found that a region of 1,485 bp upstream of *kexF*, was deleted in the genome of Nov2-2. *K. pneumoniae* possesses more potent RND-multidrug efflux systems than *E. coli*. However, we revealed that most of them did not contribute to the drug resistance of our strain at basic levels of expression. On the other hand, it was also noted that the overexpression of these pumps could lead to multidrug resistance based on exposure to antimicrobial chemicals. We conclude that these pumps may have a role to maintain the intrinsic resistance of *K. pneumoniae* when they are overexpressed. The antimicrobial chemicals selected many resistant mutants at the same minimum inhibitory concentration (MIC) or a concentration slightly higher than the MIC. These results support the importance of using antibiotics at appropriate concentrations at clinical sites.

## Introduction

*Klebsiella pneumoniae* is an important pathogen that causes urinary tract infection, opportunistic infection, and nosocomial infection. This bacterium can be isolated from not only the mammalian intestine but also environment^1,2^. It belongs to the same Enterobacteriaceae as *Citrobacter* and *Enterobacter*. Recently, antibiotic resistance levels of these bacteria are increasing, and emerging multidrug-resistant bacteria are a serious problem at clinical sites.

Multidrug efflux pumps are one of the mechanisms causing elevated resistance against many kinds of antimicrobial chemicals, such as antibiotics, dyes, antiseptics, and detergents in bacteria. So far, AcrAB^3,4^ OqxAB^5^, EefAB^6^, KexD^7^, KmrA^8^, KdeA^9^, and CepA^3^ have been reported as multidrug efflux pumps in *K. pneumoniae*. Of them, AcrAB, OqxAB, EefAB, and KexD are classified into the RND family, which is often the most important group of multidrug efflux pumps to protect against harmful chemicals in Gram-negatives. It was revealed that RND-type multidrug efflux pumps function with three components: inner membrane protein, periplasmic protein, and outer membrane protein^10^. Inner membrane protein possesses a multisite binding pocket and is able to recognize various chemicals as its substrate^11^. This protein forms a trimeric complex, and the status of each component changes into access, binding, and extrusion in order to expel substrates^11–14^. The RND-type efflux pump is also thought to play a physiological role such as in colonization of the intestinal tract and release of pathogenic factors, in addition to its role in intrinsic resistance to antibiotics and antiseptics^6,15,16^.^,^^16^.

The expression of some RND-type efflux pumps cannot be detected under laboratory culture conditions^7,17^. The expression of such pumps often increases to drive multidrug-resistant characteristics in the presence of mutations^17,18^. In *K. pneumoniae*, there are reports of clinical isolates overexpressing an RND-type efflux pump^19–22^. Most of them were reports describing overexpression of AcrB, the best-known RND in *K. pneumoniae*, but this bacterium possesses genes encoding other RNDs like OqxAB, EefAB, and KexD. The risk of such RNDs has been insufficiently assessed, even though RNDs are putative risk factors that may drive multidrug resistance by mutation(s).

Here, we isolated various drug-resistant mutants and selected strains to investigate the relation between the occurrence of multidrug-resistant mutants and RNDs. Exposure to antimicrobials produced super multidrug-resistant strains in one step.

## Results

### Isolation of mutants

*Klebsiella pneumoniae* ATCC 10031 shows hypersensitivity to various antibiotics and this strain is suitable to detect changes in levels of drug resistance. We utilized this strain to select drug-resistant mutants. The mutants were isolated in the presence of kanamycin, cefotaxime, oxacillin, or norfloxacin. They are a typical antibiotic from each category of aminoglycosides, cephems, β-lactams (not cephems), and quinolones, respectively. We also used novobiocin and ethidium to select mutants because we knew they tended to cause more mutants based on experience. As a result, 32 mutants were isolated from kanamycin-containing plates; 32 mutants were isolated from cefotaxime-containing plates; 34 mutants were isolated from oxacillin-containing plates; 49 mutants were isolated from norfloxacin-containing plates; 505 mutants were isolated from novobiocin-containing plates; 76 mutants were isolated from ethidium Br-containing plates (Table 1). The frequency isolating of mutants was 10^−7^–10^−9^, and this was considered to be in the range of the general frequency of mutant isolation.

**Table 1:** Isolated mutants at different concentrations of antimicrobial agents and the mutation frequencies.

Antimicrobial agent	MIC in ATCC10031 (μg/ml)	Number of mutants at each concentration of antimicrobial agent^a^						Total number of mutants^b^	Frequency^b^
		× 1	× 2	× 4	× 8	× 16	× 32		
Kanamycin	1	+++	32	0	n.t.	n.t.	n.t.	32	7.5 × 10^−9^
Cefotaxime	0.015	+++	32	0	n.t.	n.t.	n.t.	32	3.4 × 10^−9^
Oxacillin	32	30	3	1	0	0	n.t.	34	4.5 × 10^−9^
Norfloxacin	0.03	48	0	1	0	0	n.t.	49	4.0 × 10^−9^
Novobiocin	1	260	180	49	15	1	0	505	5.7 × 10^−9^
Ethidium Br	32	61	10	2	3	0	0	76	1.5 × 10^−7^

+++: Cells grew on the whole surface, n.t.: not tested.

^a^The concentration of an antimicrobial agent was determined with the MIC value of each antimicrobial agent in *K. pneumoniae* ATCC10031.

^b^The number of mutants and mutation frequency were calculated with colonies countable on plates.

Then, isolated mutants were tested on L-plates containing erythromycin (8 μg/ml), ethidium Br (64 μg/ml), tetraphenylphosphonium Cl (TPP Cl) (128 μg/ml), and tetracycline (4 μg/ml) to assess whether they showed the multidrug-resistant phenotype. We chose 285 strains from 505 mutants from novobiocin plates and investigated whether they showed multidrug resistance. One oxacillin-resistant mutant, 16 novobiocin-resistant mutants, and 68 ethidium Br-resistant mutants showed elevated resistance to more than two chemicals. In kanamycin-resistant mutants, cefotaxime-resistant mutants, and norfloxacin-resistant mutants, only the resistance level for the same chemical used for mutant selection was increased (Table 2).

**Table 2:** Isolation frequency of multidrug resistance in mutants.

Antimicrobial agent	Investigated mutants on agar plate	Multidrug-resistant mutants (%)*	Number of strains measured MIC	Mutants that showed marked multidrug resistance
Kanamycin	32	0 (0%)	0	0
Cefotaxime	32	0 (0%)	0	0
Oxacillin	34	1 (2.9%)	1	1
Norfloxacin	49	0 (0%)	0	0
Novobiocin	285	16 (5.6%)	8	2
Ethidium Br	76	68 (89.5%)	14	2

*The rate of multidrug-resistant mutants in investigated strains.

### Minimum inhibitory concentrations in isolated mutants

Minimum inhibitory concentrations (MICs) of antimicrobial chemicals were measured in a part of the mutants, based on resistance to several chemicals on plates. MICs of eight chemicals were measured in nine strains chosen at random from 68 that were isolated from ethidium Br plates (Table S1). All mutants showed increased MIC of TPP Cl. Of those, EB256-1 and EB256-2 showed high MIC of TPP Cl. The strain EB256-2 was also resistant to norfloxacin (Table S1). We randomly chose eight of sixteen strains resistant to novobiocin (Table S2). The MIC of four kinds of chemicals were increased more than four times in two mutants (Nov1-8 and Nov2-2) (Table 3, Table S2). The oxacillin-resistant mutant (OX128) showed elevated MICs of norfloxacin, sodium dodecyl sulfate (SDS), and ethidium Br compared with the parental strain, ATCC 10031 (Table 3). The MIC of cloxacillin was also increased in this strain (Table 3).

**Table 3:** Minimum inhibitory concentration of antimicrobial agents in isolated mutants.

Antimicrobial agent	Minimum inhibitory concentration (μg/ml)					
	ATCC10031	EB256-1	EB256-2	Nov1-8	Nov2-2	OX128
Erythromycin	8	16	16	4	256	4
Oleandomycin	16	64	64	64	> 1,024	16
Rokitamycin	8	16	16	16	64	8
Gentamicin	0.25	0.25	0.25	0.25	0.5	0.5
Kanamycin	1	1	1	1	1	1
Novobiocin	1	4	4	32	8	2
Acriflavine	4	16	16	16	32	16
Tetracycline	1	1	1	1	4	1
Cloxacillin	8	16	16	128	64	128
Norfloxacin	0.03	0.03	0.25	0.03	0.06	0.25
Chloramphenicol	1	1	1	1	1	1
Benzalkonium Cl	4	4	4	8	16	16
SDS	256	256	256	512	1,024	512
Ethidium Br	32	256	256	256	512	128
Rhodamine 6G	32	256	256	256	1,024	256
TPPCl	64	512	512	256	> 1,024	128
CTAB	16	16	16	32	32	32
Na Cholate	16,000	16,000	16,000	16,000	16,000	16,000

*CTAB* cetyltrimethylammonium bromide

Next, we measured MICs of more kinds of antimicrobial chemicals in the five strains (EB256-1, EB256-2, Nov1-8, Nov2-2, and OX128) (Table 3). These five mutants showed higher MICs for acriflavine, ethidium Br, rhodamine 6G, and TPP Cl than the parental strain.

### Ethidium efflux assay

**Figure 1:**
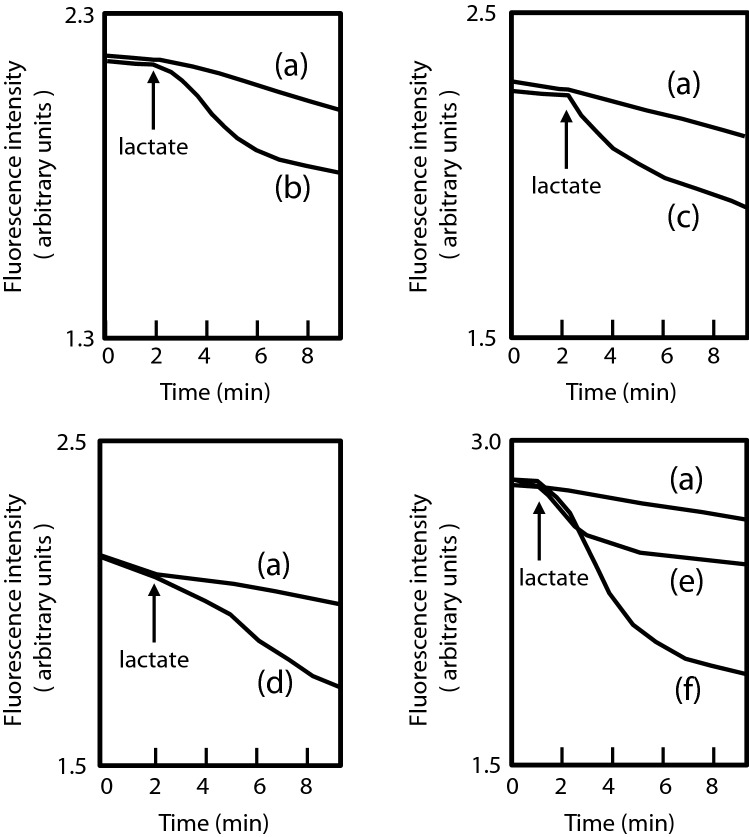
Ethidium efflux activity in the multidrug-resistant mutants. A total of 20 mM K-lactate was added to the cell suspension at the time indicated by the arrow. (**a**) ATCC 10031, (**b**) EB256-1, (**c**) EB256-2, (**d**) Nov1-8, (**e**) Nov2-2, and (**f**) OX128.

We empirically knew that drug efflux pumps play an important role in resistance to macrolides and dyes such as ethidium, rhodamine, and acriflavine. Then, we measured the efflux activity of ethidium in isolated mutants (EB256-1, EB256-2, Nov1-8, Nov2-2, and OX128) (Fig. 1). All of these mutants extruded ethidium stronger than their parental strain, ATCC 10031. This suggests that multidrug efflux pumps contribute to the increased drug resistance in these strains.

### Prediction of RND-type multidrug efflux pump in *Klebsiella pneumoniae* genome

RND-type multidrug efflux pumps are generally the most important for intrinsic resistance to various harmful chemicals in Gram negatives. Such efflux pumps, showing extremely wide substrate specificity, have been mostly classified into the RND-type in Gram-negative bacteria to date^4,23,24^. For the reasons noted above, we hypothesized that RND-type pumps lead to the multidrug-resistant phenotype in isolated mutants.

We predicted RND-type multidrug efflux pumps based on information on the genome of *K. pneumoniae* MGH78578, whose whole sequence has been identified (ACCESSION NC_009648).

Amino acid sequences of AcrB, AcrD, AcrF, MdtB, MdtC, YhiV, and CusB from *E. coli* were referred in order to predict RNDs. Predicted proteins that showed more than 40% identity were chosen. Then, it was confirmed whether the length of the amino acid residues in the predicted proteins was around 1,000 and the similarity to the reference protein was overall and not partial. Twelve genes for RND-type multidrug efflux pumps were identified in *K. pneumoniae* (KPN_RS15920 (*oqxB*), KPN_RS19875, KPN_RS25540 (*cusB*), KPN_RS11120 (*kexD*), KPN_RS21805 (*eefB*), KPN_RS11560, KPN_RS02355 (*acrB*), KPN_RS04245, KPN_RS13595, KPN_RS13600, KPN_RS15040, KPN_RS03035) (Table S3)^25^. Of those, KPN_RS15920 (*oqxB*)^26^, KPN_RS25540 (*cusB*)^27^, KPN_RS11120 (*kexD*)^7^, KPN_RS21805 (*eefB*)^6^, and KPN_RS02355 (*acrB*)^4^ were previously reported.

The RND-type multidrug efflux pump functions with three components, and each component localizes in the inner membrane, periplasmic space, and outer membrane^10^. Genes of the components of the inner membrane proteins and periplasmic proteins are often encoded in an operon. KPN_RS19875, KPN_RS11560, KPN_RS04245, KPN_RS13595, KPN_RS13600, and KPN_RS03035 possessed a putative gene for the periplasmic component upstream, but KPN_RS15040 did not have an adjacent gene for the periplasmic component like *kexD*^7^. KPN_RS13595 and KPN_RS13600 were considered to be orthologs of *mdtB* and *mdtC* from *E. coli*, respectively^28,29^. They were suggested to form an operon with KPN_RS13590, probably encoding a periplasmic component, and it was considered that the products of KPN_RS13590, KPN_RS13595, and KPN_RS13600 functioned together as one system like MdtABC. Therefore, we hypothesized that there were 11 RND-type drug efflux systems in *K. pneumoniae* MGH78578, although 12 genes for inner membrane components were in the genome of this strain (Table S3).

We named KPN_RS19870-KPN_RS19875 *kexEF*, KPN_RS11565-KPN_RS11560 *kexJK*, KPN_RS04250-KPN_RS04245 *kexSR*, and KPN_RS15040 *kexC*, respectively. KPN_RS13590-KPN_RS13595-KPN_RS13600, considered to be *mdtABC* orthologs, were named *kexVWX*, respectively (Table S3).

### Gene cloning of deduced RND-type multidrug efflux pumps

The characterization of several RND-type multidrug efflux pumps from *K. pneumoniae* were previously reported^7, 19,27^. However, these reports were of studies that investigated different conditions performed by different groups. To compare the drug-resistant pattern in the isolated resistant mutants to the substrate specificity of each RND-type pump, we cloned all deduced genes encoding RND-type efflux pumps from *K. pneumoniae* MGH78578 downstream of the *lac* promoter. Then, *E. coli* (KAM32 or KAM33) and *K. pneumoniae* ATCC 10031 were transformed with the plasmids. The strains *E. coli* KAM32 and KAM33, derivatives of TG1, lack *acrB* and *acrAB*, respectively. Then, we measured the MICs of antibiotics, antiseptics, and antimicrobial chemicals in these transformants (Table 4, Table S4). A plasmid carrying *kexC* or *kexD* was introduced into KAM32 because they were considered to need the periplasmic component, AcrA, from the host for their function.

**Table 4:** MIC of various antimicrobial agents in *E. coli* cells transformed with deduced RND-type efflux pump gene from *K. pneumoniae.*

Antimicrobial agent	Minimum inhibitory concentration (μg/ml)												
	Host: *E. coli* KAM33										Host: *E. coli* KAM32		
	pSTV28	pKAC28M	pKAB28	pKEF28	pKGH28	pKJK28	pKLM28	pKRS28	pKTU28	pKVWX28	pSTV28	pKC28	pKD28
	vector	*acrAB*	*oqxAB*	*kexEF*	*eefAB*	*kexJK*	*cusAB*	*kexRS*	*kexTU*	*kexVWX*	vector	*kexC*	*kexD*
Oxacillin	8	1,024	> 1,024	256	256	8	8	8	8	32	16	512	n.t.
Cloxacillin	4	512	64	64	128	4	4	4	4	128	4	32	4
Norfloxacin	0.03	0.25	0.5	0.03	0.125	0.03	0.03	0.03	0.03	0.03	0.03	0.03	0.03
Erythromycin	4	512	4	64	64	4	4	4	4	4	4	4	128
Kanamycin	1	1	1	1	1	1	1	1	1	1	1	1	1
Tetracycline	0.5	4	0.5	2	4	0.5	0.5	0.5	0.5	0.5	0.5	0.5	2
Novobiocin	1	512	8	64	32	1	2	2	1	8	2	8	8
Acriflavine	8	128	64	16	128	8	8	8	8	16	2	2	8
Benzalkonium Cl	4	32	32	8	16	4	4	4	4	4	4	4	8
Hoechst33342	0.5	8	4	4	> 16	0.5	0.5	0.5	0.5	0.5	0.5	0.5	1
Ethidium Br	4	1,024	128	128	1,024	4	4	4	4	4	4	4	32
Rhodamine 6G	8	> 1,024	512	256	1,024	8	8	8	8	8	8	8	32
SDS	64	> 1,024	> 1,024	> 1,024	> 1,024	512	64	64	64	64	64	> 1,024	256
TPPCl	8	1,024	512	256	1,024	8	8	8	8	8	8	8	256
Cholate	5,000	> 40,000	5,000	20,000	10,000	5,000	10,000	5,000	5,000	20,000	10,000	40,000	10,000

*n.t.* not tested.

AcrAB, OqxAB, and EefAB showed increased levels of resistance to various chemicals as expected. It was also revealed that KexEF showed highly elevated resistance to a wide variety of chemicals like AcrAB, OqxAB, and EefAB (Table 4). KexD also showed an increased level of resistance to macrolides and dyes and this result was consistent with our previous report^7^. KexC and KexVWX increased the MICs of multiple chemicals, although the number of substrates was less than for AcrAB. The MIC of SDS was elevated in KAM33/pKJK28 but not in ATCC 10031/pKJK28. The MICs of novobiocin in KAM33 with pKLM28 or pKRS28 were twice that of the control, but the difference was slight.

The MICs of the antimicrobials were also measured in *K. pneumoniae* ATCC 10031 transformed with these plasmids (Table S4). When *K. pneumoniae* was used as the host strain, its substrate specificity pattern was mostly similar to that in the *E. coli* host. However, MICs of more chemicals were increased in KAM32/pKC28 than in ATCC 10031/pKC28. The sensitivity to antimicrobials as a host cell or the compatibility of KexC and other RND components may be related to this phenomenon.

### Prediction of the drug efflux pump promoting multidrug resistance

The elevated resistance patterns of multidrug-resistant mutants were compared with the substrate specificity of each RND system in the transformants. The five strongest RND-systems (AcrAB, KexAB, KexD, KexEF, and EefABC) were considered to be related to the change of phenotype. However, the substrate specificities of these pumps overlapped and it was difficult to narrow the search down to one pump based on the substrate specificity of RNDs.

We previously reported a nonsense mutation in *acrB* in ATCC 10031^30^. This nonsense mutation was present in all mutant genomes (data not shown). Therefore, it was thought that the increased resistance of mutants was caused by powerful pumps other than the AcrAB system.

**Figure 2:**
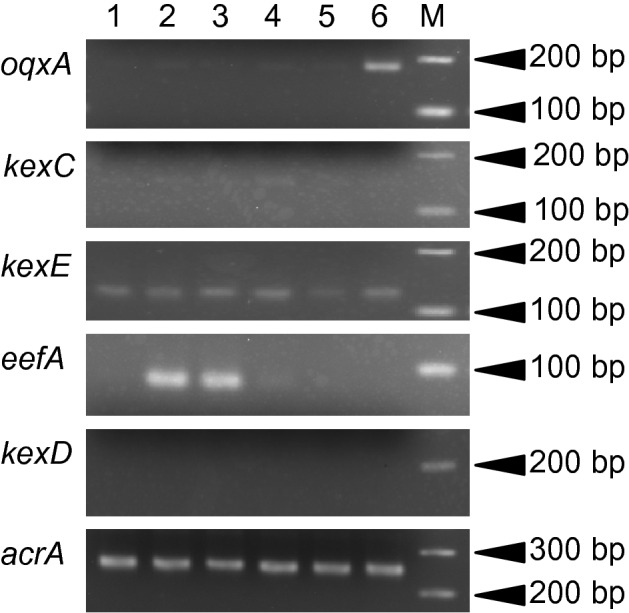
mRNA expression of *oqxA*, *kexC*, *kexE*, *eefA*, *kexD*, and *acrA* among the multidrug-resistant mutants. Lane 1: ATCC 10031, Lane 2: EB256-1, Lane 3:EB256-2, Lane 4: Nov1-8, Lane 5: Nov2-2, Lane 6: OX128, M: size marker. The expression of *acrA* was used as a control. The length of each product and reaction cycle were: *oqxA*, 179 bp, 27 cycles; *kexC*, 141 bp, 30 cycles; *kexE*, 117 bp, 29 cycles; *eefA*, 81 bp, 28 cycles; *kexD*, 184 bp, 30 cycles; *acrA*, 257 bp, 28 cycles. Quick-Load Purple 100 bp DNA Ladder (New England Biolabs Japan Inc.) was used as a size marker.

Then, we investigated the mRNA expression of *oqxA*, *kexC*, *kexE*, *eefA,* and *kexD* (Fig. 2). The expression of *eefA* was clearly increased in the mutants EB256-1 and EB256-2. The mutant OX128 showed overexpression of *oqxA*. The first report of *oqxAB* was from a plasmid in *E. coli* in 2004^35^. An ortholog of *oqxAB* was not found in the *E. coli* genome, but was found in the *K. pneumoniae* genome^36^. Several transcriptional regulator genes were located in the flanking regions of *oqxAB* in the *K. pneumoniae* genome, and it was also reported that both *rarA*, located upstream of *oqxAB,* and *oqxR*, downstream of *oqxAB,* were regulator genes of *oqxAB*^5,37^. We determined the DNA sequence of *oqxAB* and its flanking region in ATCC 10031 and OX128. As a result, there was no mutation in *rarA* or *oqxR.* However, a single substitution (G → T) was found in the intergenic region between *rarA* and *oqxA*. The mutation was out of the predicted promoters for *rarA* or *oqxA*. Also, this substitution did not coincide with any sites reported by Mark et al. in 2012^5^.

We could not detect changes in mRNA expressions of *oqxA*, *kexD*, *kexE*, or *eefA* in the mutant Nov1-8 or Nov2-2. However, the levels of resistance to many chemicals were elevated in Nov2-2, and we considered that any of the five RND systems would be definitely involved.

We focused on AcrAB again because of the wide substrate specificity of Nov2-2. We also knew that the nonsense mutation in ATCC 10031 was weakly suppressed and a small amount of AcrB was produced in ATCC 10031^30^. Then, we investigated if the suppression was facilitated to elevate the AcrB protein expression.

**Figure 3:**
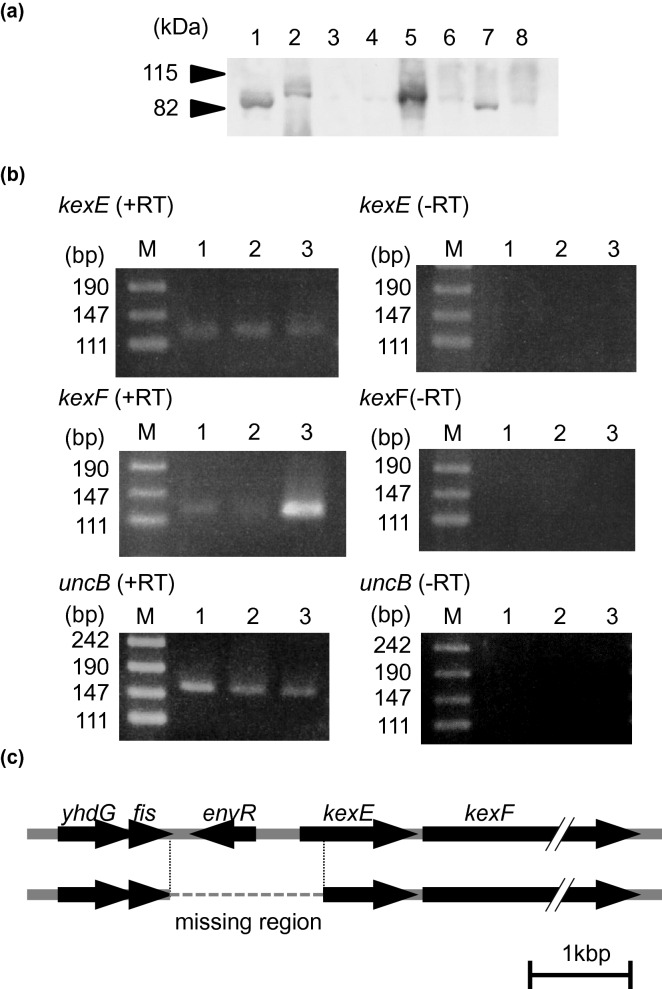
Identification of genes responsible for the increased drug resistance of the mutants. (**a**) A protein detected with anti-AcrB antibody. A protein that cross-reacted with anti-AcrB antibody of *E. coli* was detected by Western blotting. Membrane protein (20 μg) was loaded. Lane 1: Nov2-2, Lane 2: MGH78578, Lane 3: ATCC 10031, Lane 4: SKY2, Lane 5: TG1, Lane 6: KAM33, Lane 7: KAM33/pKEF28, Lane 8: KAM33/pKGH28. Lanes 1–4 were from *K. pneumoniae* and Lanes 5–8 were from *E. coli*. (**b**) mRNA expression of *kexEF* in Nov2-2. The results of reverse transcriptase reaction (RT-PCR) are shown as + RT, with –RT denoting negative controls without reverse transcription. RT-PCR for *uncB* was performed as an internal control. M: size marker, Lane 1: MGH78578, Lane 2: ATCC1003, Lane 3: Nov2-2. The annealing temperature was 50 °C for all samples. Reaction cycles were: 31 cycles for *kexE* and *kexF*, and 27 cycles for *uncB*. (**c**) Determination of the deleted region in the Nov2-2 genome. The length of the deleted region in the Nov2-2 genome was 1,485 bp, which included a part of *fis*, a whole *envR* (KPN_RS19865), the deduced promoter region of *kexEF*, and part of *kexE*.

A strong reaction signal for anti-AcrB antibody was detected in Nov2-2, but this protein was slightly smaller than AcrB (Fig. 3a). It was indicated that the protein cross-reacting with anti-AcrB antibody but not AcrB was overexpressed in Nov2-2. Of the four inner membrane components (OqxB, KexD, KexF, and EefB), the primary structure of KexF and EefB showed marked similarity with AcrB, and we tested whether these proteins cross-react with anti-AcrB antibody. It was revealed that the antibody reacted with KexF, but not with EefB (Fig. 3a).

Increased mRNA expression of *kexF* was also detected in Nov2-2, although the expression of *kexE* was at almost the same level as in the parent (Figs. 2, 3b). Therefore, we considered that the overexpression of only KexF contributed to the multidrug resistance in Nov2-2.

Then, sequencing revealed that 1,485 bp in the upstream region of *kexF* was deleted in the genome of Nov2-2 (Fig. 3c). This deleted region included a part of *kexE*, a deduced promoter region of *kexEF*, and a deduced regulator gene (KPN_RS19865). The predicted protein of KPN_RS19865 showed high similarity with EnvR from *E. coli* (identity 92%), and KPN_RS19865 was considered to be an encoding gene of the EnvR ortholog^31^.

## Discussion

We previously reported that *K. pneumoniae* ATCC 10031 was highly sensitive to various antibiotics and antimicrobial chemicals because of a nonsense mutation in *acrB*^30^. In this study, we isolated multidrug-resistant mutants from ATCC 10031 by exposure to antimicrobial chemicals.

The multidrug-resistant mutant OX128 was isolated in the presence of oxacillin and the overexpression of *oqxAB* was clarified in OX128. There have been numerous reports of clinically isolated mutants resistant to β-lactams, and the causes of the resistance were acquisition of a β-lactamase gene or reduction of outer membrane proteins. Compared with them, reports that drug efflux pumps caused resistance to β-lactams are much fewer. Hence, it was suggested that β-lactams were not good substrates for drug efflux pumps. However, our results suggest that efflux pumps also possibly caused increased resistance to β-lactams.

We cloned all genes that were deduced to be RND-type efflux pump genes to compare with previously reported ones. In addition to *acrAB*^4,32^, *oqxAB*^5^, *eefAB*^6^, and *kexD*^7^, we noted that KexEF conferred resistance to a wide variety of antimicrobial chemicals. The substrate specificity of KexEF resembled that of EefAB as far as we investigated.

Ten kinds of RND-type multidrug efflux pump genes were cloned in *Enterobacter cloacae,* and the relationship between these pumps and drug resistance was investigated^16^. ECL_01758 reported in this article showed 97% identity with KexD. Although ECL_01758 elevated the MIC of amikacin and gentamicin, KexD did not increase the MIC of kanamycin belonging to the same aminoglycoside group. Besides, acriflavine and ethidium were good substrates for KexD, but cloned ECL_01758 did not show markedly elevated levels of resistance to these chemicals. They both were very similar proteins regarding their primary structure. It is interesting to note that the difference of a few amino acids residues between them may cause such different substrate specificities.

Overexpression of *eefA* was clearly observed in EB256-1 and EB256-2. Meanwhile, resistance to norfloxacin was different between EB256-1 and EB256-2, although both were EefAB-overexpressing mutants. The MIC of norfloxacin was increased in *K. pneumoniae* ATCC 10031/pKGHA28 and *E. coli* KAM33/pKGH28, and this result coincided with the pattern of EB256-2. Therefore, EB256-1 may possess an additional mutation that decreases quinolones resistance.

Tamae et al. reported genes showing super-sensitivity to quinolones with the Keio collection^33^, and Han et al. also reported genes that increased lethality by exposure to nalidixic acid when disrupted^34^. These studies were independently performed. It is interesting that the genes reported by each group did not overlap. This means that many genes may be related to quinolone resistance. One mutation of these genes reported by Tamae et al. or Han et al. might cause the different phenotype of quinolone resistance between EB256-1 and EB256-2.

The mRNA expression of *kexE* was observed under the laboratory culture conditions (Fig. 2) and it was also detected in Nov2-2, which lacked *kexE*. This is the reason why the primers to test *kexE* expression were designed in the region downstream of the deleted part. *kexEF* was deduced to form an operon, but it was clear that the expression of only *kexF* was markedly increased in Nov2-2. Therefore, it was suggested that the transcription initiation point of *kexF* mRNA should be downstream from the primer, kexE Fw, used to detect *kexE* expression.

In this study, we revealed that *K. pneumoniae* possessed a potent RND-type multidrug efflux pump, KexEF. KexEF increased the resistance to various antimicrobial chemicals in the host cells when the genes were cloned under *lac* promoter (Table 4 and Table S4). However, it was considered that KexEF would not play a significant role in antimicrobial resistance in ATCC 10031, at least under the laboratory culture conditions. We give two reasons for this: Firstly, antibiotic resistance levels were increased in Nov2-2, in which expression levels of *kexF* were markedly increased. Secondly, levels of resistance to antimicrobials were almost the same in *K. pneumoniae* ATCC 10031, a strain deficient in *acrB,* and *E. coli* KAM32, an *acrB*-disrupted strain. This means that KexEF is not a system equivalent to AcrAB in *K. pneumoniae,* although KexEF is a powerful multidrug efflux system. It is considered that the typical expression level of KexEF is too low to be an alternative system to AcrAB.

In Nov2-2, *kexE* was deleted and only KexF was overexpressed. Then, KexF was considered to co-work with AcrA in Nov2-2 because AcrA shows high-level similarity with KexE and is expressed at a higher level than any other periplasmic components of RND-type multidrug efflux systems. The deletion of *envR* caused overexpression of KexF in the mutant strain, Nov2-2. This result suggests that the lack of EnvR, a possible repressor of *kexEF,* and occurrence of a promoter-like sequence upstream of *kexF* caused the overexpression of KexF. EnvR might repress the expression of *kexEF* to some extent. It was reported that EnvR controlled the expression of *acrAB* more than *acrEF* adjacent to the *envR* gene in *E. coli*^31^. However, the mRNA expression of *acrA* was constant even in Nov2-2 in Fig. 2, and the repression of *acrAB* by EnvR would be absent or less effective in *K. pneumoniae*.

The plasmid carrying *kexEF* genes conferred marked resistance to various antimicrobials, and no problem of compatibility between KexE and KexF was noted. However, KexF overexpression in Nov2-2 possibly functioned with AcrA as its periplasmic component.

In this study, the resistant mutants were highly isolated at 1–4 times the MIC of each antimicrobial chemical, and this corresponds well with the mutant selection window of antibiotics used clinically. Therefore, a multidrug-resistant mutant may be generated by only a single administration of an antibiotic if a similar concentration is used.

Besides, chemicals such as pesticides are spread in fields, detergents are copiously used in daily life, and chemicals are disposed of down industrial drains in modern times. They are usually diluted to non-toxic concentrations for organisms before being released into the environment in Japan, but it is not considered whether the concentration is low enough to avoid selecting drug-resistant bacteria. Therefore, in terms of the problem of bacterial antimicrobial resistance, it may also be a considerable risk factor to release chemicals to environment even though they may be diluted to harmless levels for higher organism. Before chemicals are released into the environment, assessment of whether the concentration is low enough to avoid selecting resistant bacteria might be necessary in the future.

## Materials and methods

### Bacterial strains, plasmids, and media

The strains used in this study are listed in Table S5. The strain *K. pneumoniae* MGH78578 was a gift from Dr. Michael McClelland of the Sidney Kimmel Cancer Center in San Diego, CA, USA. The strain *K. pneumoniae* ATCC 10031 was purchased from the American Type Culture Collection. This is an *acrB*-defective strain, which shows hyper-sensitivity to various chemicals^30^. The strain *E. coli* KAM32 (Δ*acrB*, Δ*ydhE*) and KAM33 (Δ*acrAB*, Δ*ydhE*) are drug-hypersusceptible^38^. The strains *K. pneumoniae* and *E. coli* were grown aerobically in L medium (1% polypeptone, 0.5% yeast extract, and 0.5% NaCl, pH 7.0) at 37 °C. Mueller–Hinton broth was used when the minimum inhibitory concentration (MIC) was measured. Chloramphenicol (final concentration: 20 μg/ml) was added to L medium when needed. Cell growth was monitored turbidimetrically at 650 nm. L-agar plates contained 1.5% agar.

### PCR cloning of putative RND-type efflux pump genes

The coding region of each RND-type efflux pump gene was amplified by PCR. The genome was isolated from *K. pneumoniae* MGH78578 and used as a template for PCR. The resultant plasmids are listed in Table S5. The primers used in this study are listed in Table S6. The conditions for PCR were 1 min at 94 °C, 1 min at 60 °C, and 5 min at 68 °C, repeated for 35 cycles. KOD-Plus DNA polymerase (TOYOBO Co. LTD., Osaka, Japan) was used for DNA amplification. After digestion with appropriate restriction enzymes and gel purification, each DNA fragment was ligated to pSTV28, which was pre-digested with the appropriate enzyme. The absence of nucleotide substitution by PCR in ORFs was confirmed by sequencing.

### MIC determination

We previously described the method to measure minimum inhibitory concentrations (MICs) of various antimicrobial agents^7^. The same experiment was repeated at least five times and the most reproducible values are shown in Tables.

### Efflux assay for ethidium

The *K. pneumoniae* cells were grown in L medium until OD_650_≈0.7. Cells were harvested and washed twice with modified Tanaka buffer (34 mM KH_2_PO_4_, 64 mM K_2_HPO_4_, 20 mM (NH_4_)_2_SO_4_, 0.3 mM MgSO_4_, 1 μM FeSO_4_, 1 μM ZnCl_2_, 10 μM CaCl_2_, and 2 mM MgSO_4_, pH 7.0)^39^. Cells were resuspended in this buffer containing carbonyl cyanide *m*-chlorophenylhydrazone (CCCP, final concentration: 40 μM) and ethidium Br (final concentration: 10 μM). Cells were incubated at 37 °C for 0.5 h to deplete energy and ethidium was loaded into the cells during incubation. Energy-starved cells were harvested and washed twice with 20 mM MOPS (3-morpholinopropanesulfonic acid)-TMAH (tetramethylammonium hydroxide) buffer (pH 7.0) containing 2 mM MgSO_4_ and 10 μM ethidium Br. The fluorescence intensity of ethidium was measured at an excitation wavelength of 500 nm and emission wavelength of 580 nm, respectively, using the fluorescence spectrophotometer F-2000 (Hitachi Co.). Lactate (final concentration: 20 mM) was added after measurement of the baseline for 1–2 min.

### RNA preparation and reverse transcriptase-polymerase chain reaction (RT-PCR) analysis

The method of RNA preparation from *K. pneumoniae* was previously described^7^. The extracted RNA was applied to RT-PCR with the QIAGEN One-Step RT-PCR Kit (QIAGEN K.K., Japan). The primers used for RT-PCR are listed in Table S6. PCR without a reverse-transcriptase reaction was performed to confirm the absence of detectable DNA contamination in the extracted RNA solution.

### Western blotting

Everted membrane vesicles was prepared by the method described previously^30^. Details of the method for Western blotting were also described previously^30^. The anti-AcrB antibody to *E. coli* was used as a primary antibody, which also cross-reacted with AcrB from *K. pneumoniae*^30^*.* This antibody was provided by Dr. A. Yamaguchi (Institute of Scientific and Industrial Research, Osaka University). The secondary antibody, goat anti-rabbit IgG with horseradish peroxidase, was purchased from Thermo Fisher Scientific K.K.. The ECL Western blotting detection system (GE Healthcare UK Ltd., Buckinghamshire, England) was used for detection.

## Supplementary information

Supplementary file1 (PPTX 1328 kb)

Supplementary file2 (DOCX 78 kb)
