# Supplementary material for: The role of RND-type efflux pumps in multidrug-resistant mutants of Klebsiella pneumoniae
Source: Sci Rep. 2020 Jul 2;10:10876. doi: 10.1038/s41598-020-67820-x (PMC7331594; doi:10.1038/s41598-020-67820-x)
Supplement: Supplementary file 1 — Supplementary file1 (PPTX 1328 kb) [file 41598_2020_67820_MOESM1_ESM.pptx]

## Slide 1
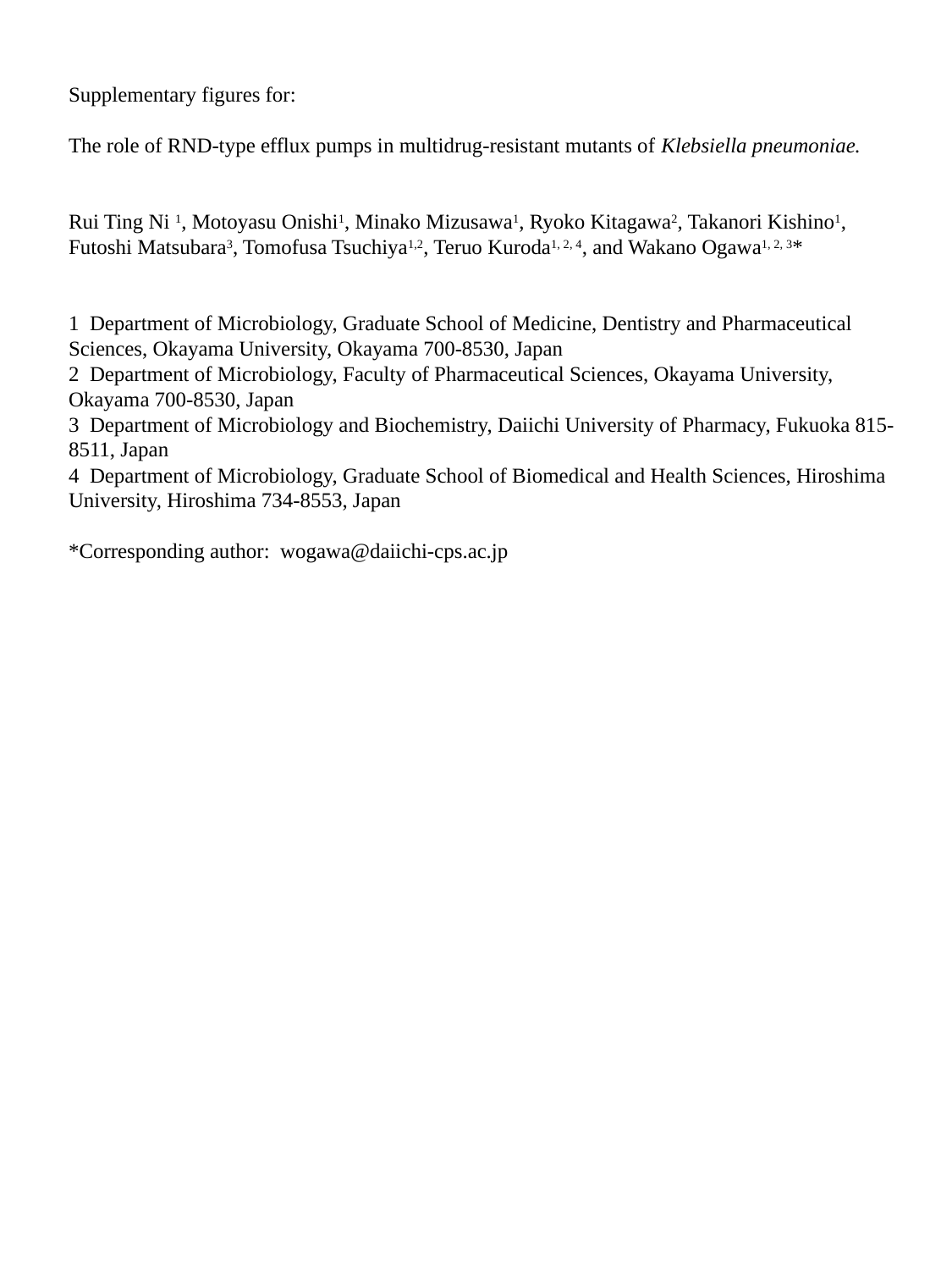

Supplementary figures for:
The role of RND-type efflux pumps in multidrug-resistant mutants of Klebsiella pneumoniae.
Rui Ting Ni 1, Motoyasu Onishi1, Minako Mizusawa1, Ryoko Kitagawa2, Takanori Kishino1, Futoshi Matsubara3, Tomofusa Tsuchiya1,2, Teruo Kuroda1, 2, 4, and Wakano Ogawa1, 2, 3*
1 Department of Microbiology, Graduate School of Medicine, Dentistry and Pharmaceutical Sciences, Okayama University, Okayama 700-8530, Japan
2 Department of Microbiology, Faculty of Pharmaceutical Sciences, Okayama University, Okayama 700-8530, Japan
3 Department of Microbiology and Biochemistry, Daiichi University of Pharmacy, Fukuoka 815-8511, Japan
4 Department of Microbiology, Graduate School of Biomedical and Health Sciences, Hiroshima University, Hiroshima 734-8553, Japan
*Corresponding author: wogawa@daiichi-cps.ac.jp

## Slide 2
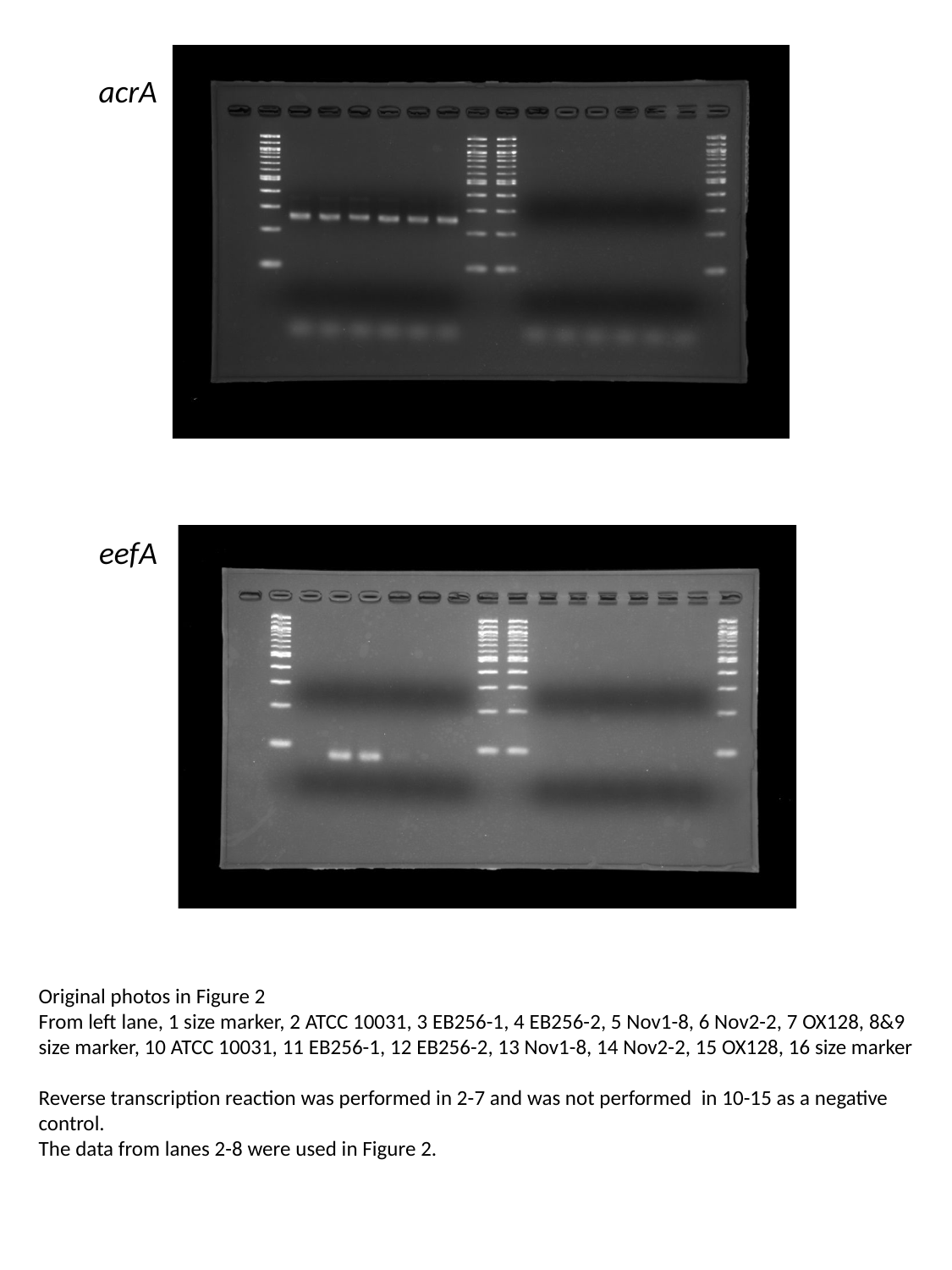

acrA
eefA
Original photos in Figure 2
From left lane, 1 size marker, 2 ATCC 10031, 3 EB256-1, 4 EB256-2, 5 Nov1-8, 6 Nov2-2, 7 OX128, 8&9 size marker, 10 ATCC 10031, 11 EB256-1, 12 EB256-2, 13 Nov1-8, 14 Nov2-2, 15 OX128, 16 size marker
Reverse transcription reaction was performed in 2-7 and was not performed in 10-15 as a negative control.
The data from lanes 2-8 were used in Figure 2.

## Slide 3
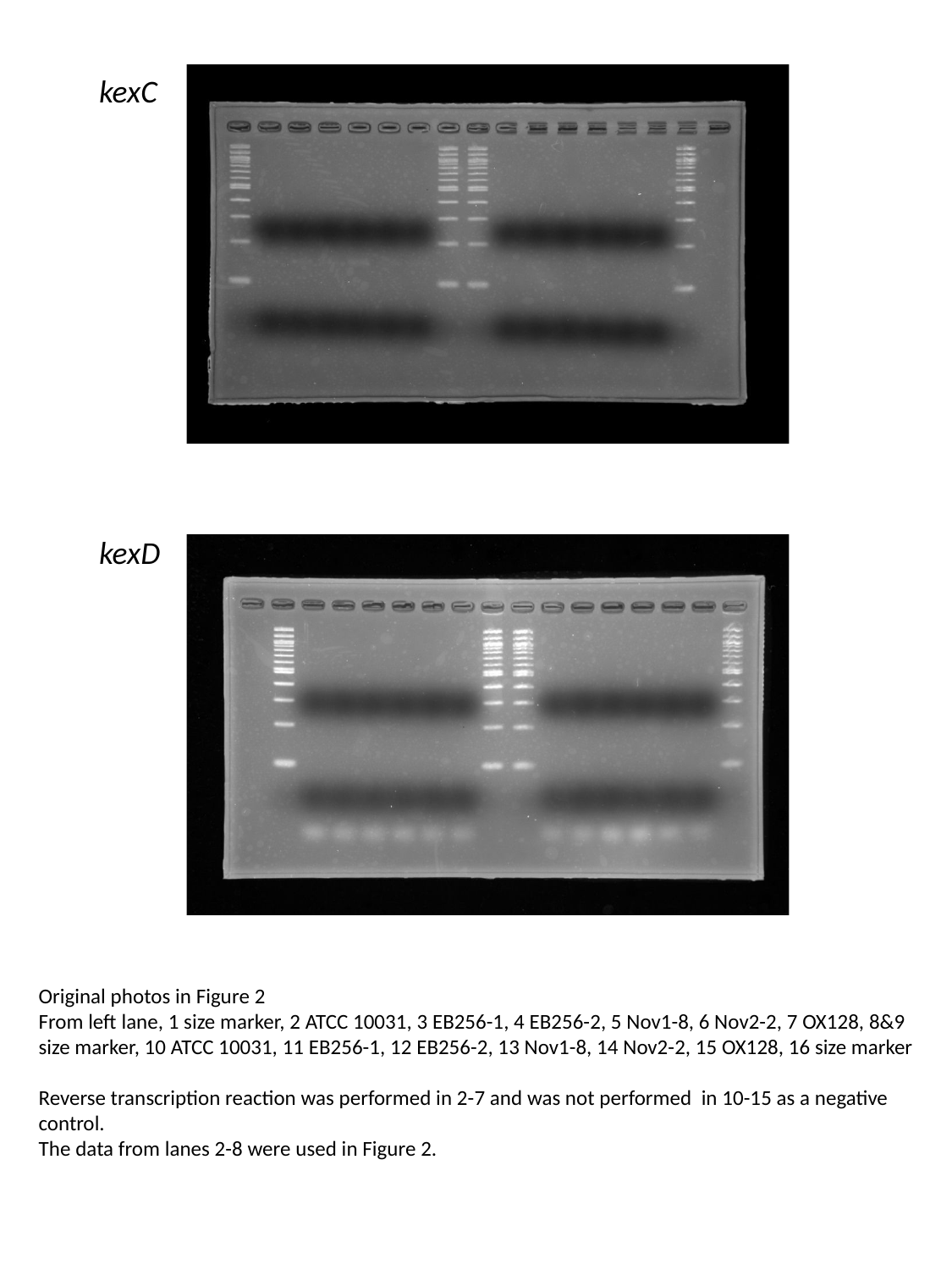

kexC
kexD
Original photos in Figure 2
From left lane, 1 size marker, 2 ATCC 10031, 3 EB256-1, 4 EB256-2, 5 Nov1-8, 6 Nov2-2, 7 OX128, 8&9 size marker, 10 ATCC 10031, 11 EB256-1, 12 EB256-2, 13 Nov1-8, 14 Nov2-2, 15 OX128, 16 size marker
Reverse transcription reaction was performed in 2-7 and was not performed in 10-15 as a negative control.
The data from lanes 2-8 were used in Figure 2.

## Slide 4
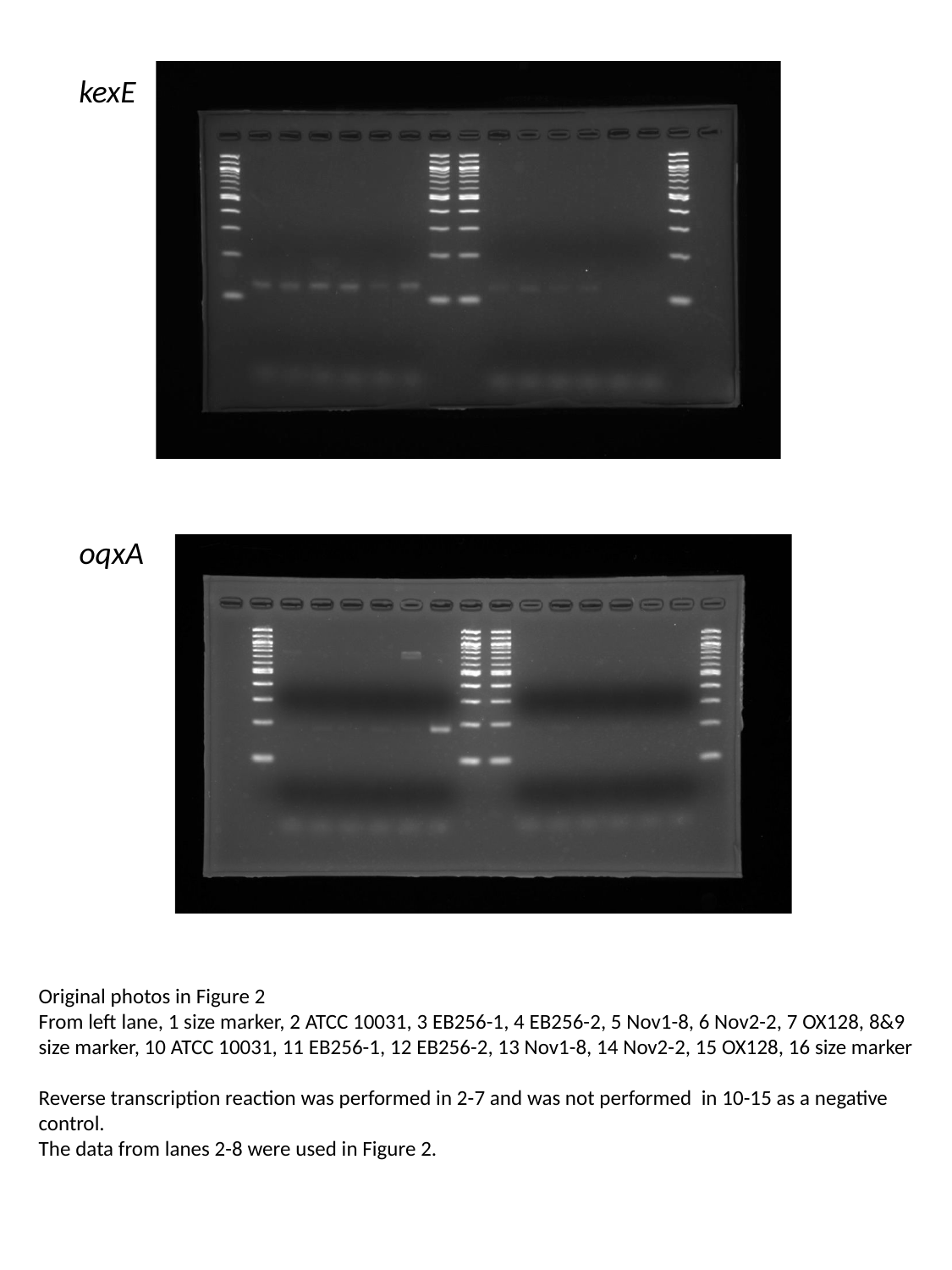

kexE
oqxA
Original photos in Figure 2
From left lane, 1 size marker, 2 ATCC 10031, 3 EB256-1, 4 EB256-2, 5 Nov1-8, 6 Nov2-2, 7 OX128, 8&9 size marker, 10 ATCC 10031, 11 EB256-1, 12 EB256-2, 13 Nov1-8, 14 Nov2-2, 15 OX128, 16 size marker
Reverse transcription reaction was performed in 2-7 and was not performed in 10-15 as a negative control.
The data from lanes 2-8 were used in Figure 2.

## Slide 5
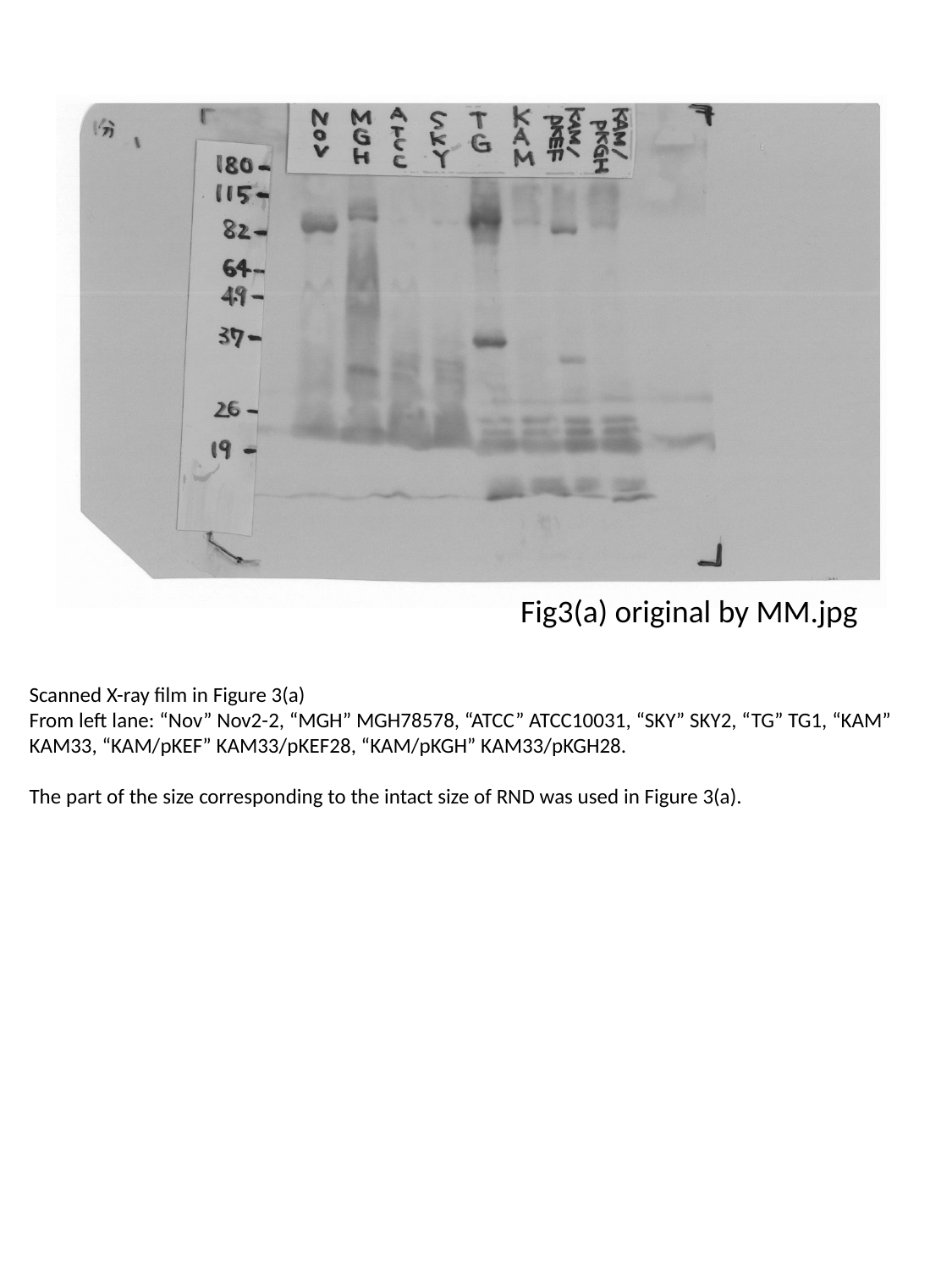

Fig3(a) original by MM.jpg
Scanned X-ray film in Figure 3(a)
From left lane: “Nov” Nov2-2, “MGH” MGH78578, “ATCC” ATCC10031, “SKY” SKY2, “TG” TG1, “KAM” KAM33, “KAM/pKEF” KAM33/pKEF28, “KAM/pKGH” KAM33/pKGH28.
The part of the size corresponding to the intact size of RND was used in Figure 3(a).

## Slide 6
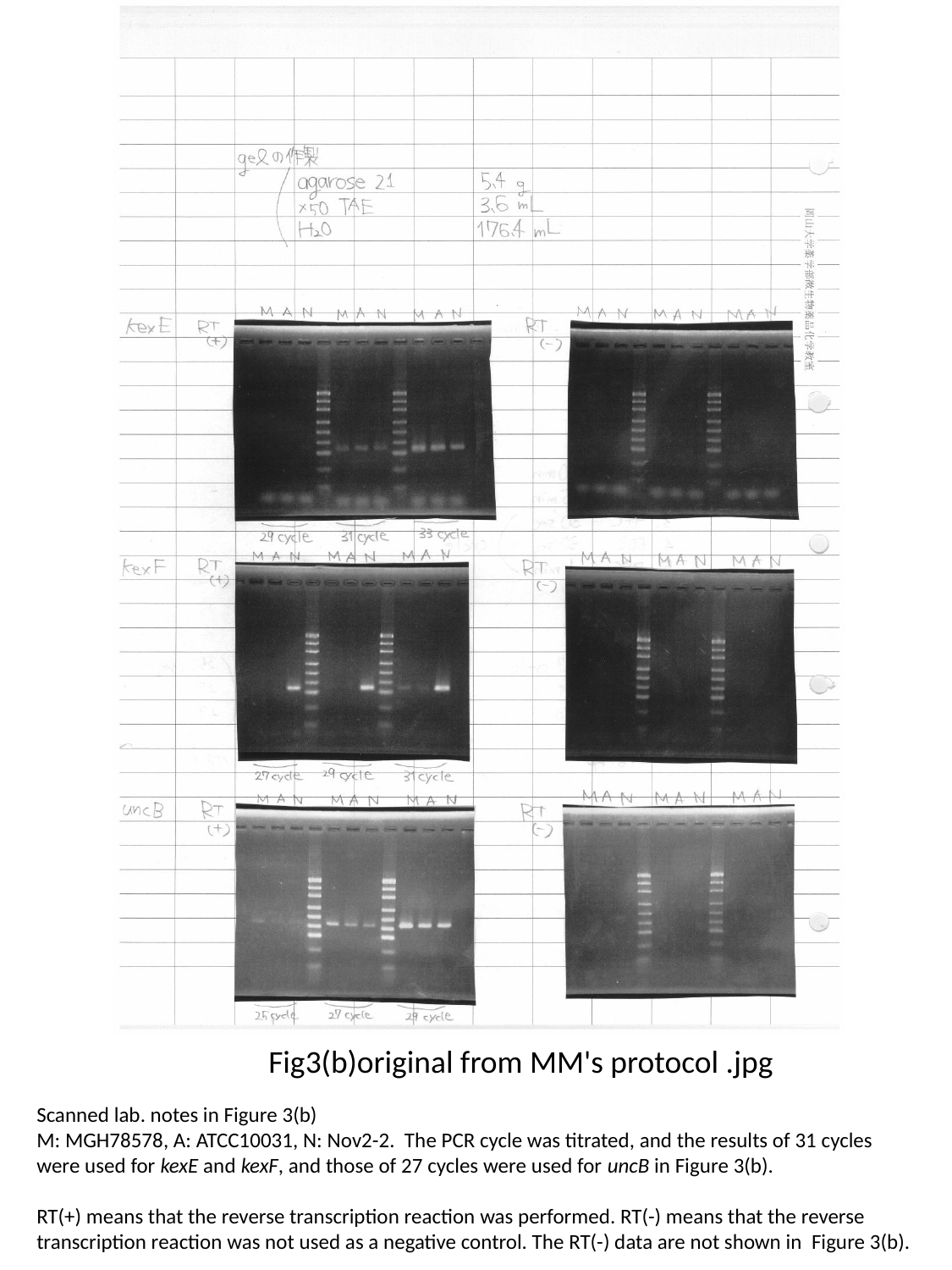

Fig3(b)original from MM's protocol .jpg
Scanned lab. notes in Figure 3(b)
M: MGH78578, A: ATCC10031, N: Nov2-2. The PCR cycle was titrated, and the results of 31 cycles were used for kexE and kexF, and those of 27 cycles were used for uncB in Figure 3(b).
RT(+) means that the reverse transcription reaction was performed. RT(-) means that the reverse transcription reaction was not used as a negative control. The RT(-) data are not shown in Figure 3(b).
