# Supplementary material for: The role of RND-type efflux pumps in multidrug-resistant mutants of Klebsiella pneumoniae
Source: Sci Rep. 2020 Jul 2;10:10876. doi: 10.1038/s41598-020-67820-x (PMC7331594; doi:10.1038/s41598-020-67820-x)
Supplement: Supplementary file 2 — Supplementary file2 (DOCX 78 kb) [file 41598_2020_67820_MOESM2_ESM.docx]

Supplementary tables for:

The role of RND-type efflux pumps in multidrug-resistant mutants of *Klebsiella pneumoniae.*

Rui Ting Ni ^1^, Motoyasu Onishi^1^, Minako Mizusawa^1^, Ryoko Kitagawa^2^, Takanori Kishino^1^, Futoshi Matsubara^3^, Tomofusa Tsuchiya^1,2^, Teruo Kuroda^1, 2, 4^, and Wakano Ogawa^1, 2, 3^*

1 Department of Microbiology, Graduate School of Medicine, Dentistry and Pharmaceutical Sciences, Okayama University, Okayama 700-8530, Japan

2 Department of Microbiology, Faculty of Pharmaceutical Sciences, Okayama University, Okayama 700-8530, Japan

3 Department of Microbiology and Biochemistry, Daiichi University of Pharmacy, Fukuoka 815-8511, Japan

4 Department of Microbiology, Graduate School of Biomedical and Health Sciences, Hiroshima University, Hiroshima 734-8553, Japan

*Corresponding author: wogawa@daiichi-cps.ac.jp

Table S1 MICs of antimicrobial chemicals in ethidium mutants

ATCC10031 EB32-1 EB32-2 EB32-4 EB32-5 EB32-6 EB32-7 EB32-8 EB256-1 EB256-2

Norfloxacin 0.03 0.03 0.06 0.03 0.03 0.03 0.03 0.03 0.03 0.25

Erythromycin 8 8 8 8 8 8 8 8 16 16

Tetracycline 1 1 1 1 1 1 1 1 1 1

Kanamycin 0.5 0.5 0.5 0.5 0.5 0.5 0.5 0.5 1 1

Chloramphenicol 1 1 1 1 1 1 1 1 1 1

Ethidium Br 32 256 128 256 256 128 128 128 256 256

TPP Cl 32 128 256 128 128 128 128 128 512 512

SDS 128 256 256 256 256 256 256 256 256 256

Table S2 MICs of antimicrobial chemicals in novobiocin mutants

ATCC10031 Nov 1-8 Nov 2-2 Nov2-3 Nov 2-19 Nov 2-20 Nov 2-22 Nov 2-23 Nov2-48 Norfloxacin 0.03 0.03 0.06 0.06 0.06 0.06 0.03 0.06 0.06

Erythromycin 8 4 256 8 8 16 8 16 8

Tetracycline 1 1 4 1 1 1 1 1 1

Kanamycin 1 1 1 4 4 4 4 4 4

Chloramphenicol 1 1 1 1 1 1 1 1 1

Cloxacillin 8 128 64 4 8 16 4 4 64

Oleandomycin 16 64 >1024 16 16 16 16 16 16

Ethidium Br 32 256 512 32 32 32 16 16 16

TPP Cl 64 256 >1024 64 64 64 64 64 32

SDS 256 512 1024 128 256 128 128 128 128

Novobiocin 1 32 8 8 2 8 8 8 4

Table S3 Deduced RND-type drug efflux systems in the genome of *Klebsiella pneumoniae* MGH78578

No. of No. of RND Direction KPN number Comment

RND-system & Gene

*oqxA* plus KPN_RS15915 Putative ortholog of *oqxA* on pOLA52 from *E. coli*,

periplasmic protein

I

1 *oqxB* plus KPN_RS15920 Putative ortholog of *oqxB* on pOLA52 from *E. coli*,

inner membrane protein

*kexE* plus KPN_RS19870 Putative periplasmic protein

II

2 *kexF* plus KPN_RS19875 Putative inner membrane protein

*cusA* minus KPN_RS25535 Putative ortholog of *cusA* from *E. coli*, periplasmic protein

3 *cusB* minus KPN_RS25540 Putative ortholog of *cusB* from *E. coli*, inner membrane protein

III

*cusF* minus KPN_RS25545 Putative ortholog of *cusF* from *E. coli*, binding protein

*cusC* minus KPN_RS25550 Probable ortholog of *cusC* from *E. coli,* outer membrane protein

IV

4 *kexD* minus KPN_RS11120 multidrug efflux pump, inner membrane protein

*eefA* plus KPN_RS21800 Putative ortholog of *eefA* from *Enterobacter aerogenes,*

periplasmic protein

V

5 *eefB* plus KPN_RS21805 Putative ortholog of *eefB* from *Enterobacter aerogenes*,

inner membrane protein

*eefC* plus KPN_RS21810 Putative ortholog of *eefC* from *Enterobacter aerogenes*

outer membrane protein

*kexJ* minus KPN_RS11565 Putative periplasmic protein

VI

6 *kexK* minus KPN_RS11560 Putative inner membrane protein

*acrA* minus KPN_RS02360 ortholog of *acrA* from *E. coli*, periplasmic protein

VII

7 *acrB* minus KPN_RS02355 ortholog of *acrB* from *E. coli*

*kexS* minus KPN_RS04250 Putative periplasmic protein

VIII

8 *kexR* minus KPN_RS04245 Putative inner membrane protein

*kexV* plus KPN_RS13590 Putative ortholog of *mdtA* from *E. coli*, periplasmic protein

IX

9 *kexW* plus KPN_RS13595 Putative ortholog of *mdtB* from *E. coli*, inner membrane protein

10 *kexX* plus KPN_RS13600 Putative ortholog of *mdtC* from *E. coli*, inner membrane protein

X

11 *kexC* plus KPN_RS15040 Putative ortholog of *acrD* from *E. coli*, inner membrane protein

*kexT* plus KPN_RS03030 Putative periplasmic protein

XI

12 *kexU* plus KPN_RS03035 Putative inner membrane protein

* * *kocC* plus KPN_RS18470 Compatible outer membrane component

Table S4 MICs of various antimicrobial agents in *K. pneumoniae* ATCC10031 harboring each plasmid

Minimum inhibitory concentration (μg/ml)

Antimicrobial Host: *K. pneumoniae* ATCC10031

agent pSTV28 pKAC28M pKAB28 pKC28 pKD28 pKEF28 pKGHA28 pKJK28 pKLM28 pKRS28 pKTU28 pKVWX28

Cloxacillin 8 512 256 128 16 512 256 8 8 8 8 64

Norfloxacin 0.03 0.25 　　 0.5 0.03 0.03 0.06 0.125 0.03 0.03 0.03 0.03 0.03

Erythromycin 8 1024 　　 8 8 256 256 128 8 8 　　 8 8 16

Kanamycin 1 1 　　 1 1 1 1 1 1 1 1 1 1

Tetracycline 1 2 　　 1 1 2 2 4 1 1 1 1 1

Novobiocin 4 128 　　 4 4 4 4 16 4 8 8 4 32

Acriflavine 8 256 　 128 8 16 32 128 8 8 8 8 8

Benzalkonium Cl 4 16 16 4 8 8 8 8 4 4 4 4

Hoechst33342 2 >16 16 2 8 >16 >16 2 2 2 2 2

Ethidium Br 64 >1024 　 512 64 256 1024 >1024 64 64 64 64 64

Rhodamine 6G 32 >1024 >1024 32 128 1024 512 32 32 32 32 32

SDS 256 >1024 >1024 512 256 >1024 >1024 >1024 256 256 256 256

TPPCl 128 >1024 1024 128 512 >1024 >1024 128 128 128 128 128

Cholate 10000 40000 20000 20000 20000 40000 20000 10000 10000 10000 10000 20000

Deoxycholate 2500 >40000 >40000 10000 10000 20000 20000 5000 5000 2500 2500 40000

Table S5 Strains and plasmids used in this study

Strains and plasmids Reference

*K. pneumoniae*

MGH78578 multidrug-resistant strain, used for the genome project

ATCC10031 ATCC collection, the parental strain of SKY2

SKY2 *acrAB*-disrupted strain, derivative of ATCC10031 7

*E. coli*

TG1 *supE*, *hsd*Δ5, *thi*, Δ(*lac-proAB*)/F'〔*traD*36, *proAB*^+^, *lacI*^q^, *lacZ*ΔM15〕

parental strain of KAM32 and KAM33,

KAM32 deletion mutant of *acrB* and *ydhE*, cloning host 38

KAM33 deletion mutant of *acrAB* and *ydhE* 24

Plasmids

pSTV28 vector Takara Bio, Inc.

pKAC28M pSTV28 derivative carrying PCR-cloned *acrAB* 30

pKAB28 pSTV28 derivative carrying *oqxAB* This study

pKC28 pSTV28 derivative carrying *kexC* This study

pKD28 pSTV28 derivative carrying *kexD* This study

pKEF28 pSTV28 derivative carrying *kexEF* This study

pKGH28 pSTV28 derivative carrying *eefAB* This study

pKGHA28 pSTV28 derivative carrying *eefABC* This study

pKJK28 pSTV28 derivative carrying *kexJK* This study

pKLM28 pSTV28 derivative carrying *cusAB* This study

pKRS28 pSTV28 derivative carrying *kexRS* This study

pKTU28 pSTV28 derivative carrying *kexTU* This study

pKVWX28 pSTV28 derivative carrying *kexVWX* This study

Table S6 Primers used in this study

Primers Sequences purpose

kexAB Fw GTTAGGAATTCTTCTCACGCTGCGTCTTGC cloning *oqxAB*

kexAB Re TAATAAGCTTCAGGCTAGGCGGGCAGATCC cloning *oqxAB*

kexC Fw AAGGAATTCTTCACACCGGCAAGGGATAATGC cloning *kexC*

kexC Re AAGGTCGACAATAAGCGCGCTAGGGTTCTG cloning *kexC*

kexD Fw TATGGATCCTGGAAGTACACCTGATG cloning *kexD*

kexD Re ACACTGCAGAGATTAGCCGTTC cloning *kexD*

kexEF Fw ACGCGGATCCGTACGTAATCTATAAGGAAC cloning *kexEF*

kexEF Re AAAGGATCCCAAGACATAGCCGAGTT cloning *kexEF*

kexGH Fw ATATGGATCCCGGACGTCTTATATCTTGAG cloning *eefAB*

kexGH Re GACAAAGCTTCACGTGCCATAACGATTCC cloning *eefAB*

kexJK Fw ACGATATCAATGGCCTTCAACGGAACAGTATGC cloning *kexJK*

kexJK Re AATGCATGCTGCCGAGGATATGAAGGTTACC cloning *kexJK*

kexLM Fw TATGAATTCACGGTAGGCCCCTCAACAAC cloning *kexLM*

kexLM Re GCAAGCTTACCAGGTTCAGATTCATAGGGAC cloning *kexLM*

kexRS Fw CCTGAATTCGAGGGGAATATTGTGAACCGT cloning *kexRS*

kexRS Re AAATGTCAGCAGCCCGAGGATCCAGTGATA cloning *kexRS*

kexTU Fw AAGAATTCCCGCTGCGCTACTGGTTTGTCA cloning *kexTU*

kexTU Re AAAACGAATTCTCACCCTACCCCTCTTCCA cloning *kexTU*

kexVW Fw ATTAGTACTAACGCTTCAGGATGAGACCG cloning *kexVW*

kexVW Re ACTTCACTTACGCCTCCTCTTCCTGAC cloning *kexVW*

kexWX Fw GCATCATTCTGCTGATCGGCATCGTG cloning *kexWX*

kexWX Re AACGGCATGCTGGCGGTGAAGAAGATATTG cloning *kexWX*

acrA Fw GTCCTCAGGTCAGTGGCATTA RT-PCR for *acrA*

acrA Re ATTGCTCTGCTGCGCCGTTG RT-PCR for *acrA*

oqxA F(RT) AAGGTGCTGGTGAAGTCGATC RT-PCR for *oqxA*

oqxA Re(RT) GCGCGATAGGTTCTGTCATC RT-PCR for *oqxA*

kexC Fw TGCCGGTTGAACAGTATCCC RT-PCR for *kexC*

kexC Re ACATCAGGTTATCGAGGCCG RT-PCR for *kexC*

kexD Fw ACCGGTTGCGCAATACCCTG RT-PCR for *kexD*

kexD Re CGTAATTGACGCCATCCCTG RT-PCR for *kexD*

kexE Fw TTACGCTTCAGGATGATACCG RT-PCR for *kexE*

kexE Re GGTCCATGTCGTGAACAGC RT-PCR for *kexE*

k.p-acrF(Fw)RT GGTCCATGTCGTGAACAGC RT-PCR for *kexF*

k.p-acrF(Rv)RT TTACGCTTCAGGATGATACC G RT-PCR for *kexF*

kexG Fw GCGGCATCATCCAGAAAC RT-PCR for *eefA*

kexG Re TGGAGGGATCGATCTGGTAA RT-PCR for *eefA*
